# Severing umbilical ties

**DOI:** 10.7554/eLife.63128

**Published:** 2020-10-12

**Authors:** Jessica E Wagenseil, Karen M Downs

**Affiliations:** 1 Department of Mechanical Engineering and Materials Science, Washington UniversitySt. LouisUnited States; 2 Department of Cell and Regenerative Biology, University of Wisconsin-Madison School of Medicine and Public HealthMadisonUnited States

**Keywords:** umbilical cord, proteoglycans, extracellular matrix, vascular smooth muscle, birth, vascular engineering, Human, Mouse

## Abstract

High levels of proteins called proteoglycans in the walls of umbilical arteries enable these arteries to close rapidly after birth and thus prevent blood loss in newborns.

**Related research article** Nandadasa S, Szafron JM, Pathak V, Murtada SI, Kraft CM, O'Donnell A, Norvik C, Hughes C, Caterson B, Domowicz MS, Schwartz NB, Tran-Lundmark K, Veigl M, Sedwick D, Philipson EH, Humphrey JD, Apte SS. 2020. Vascular dimorphism ensured by regulated proteoglycan dynamics favors rapid umbilical artery closure at birth. *eLife* **9**:e60683. doi: 10.7554/eLife.60683

Omphaloskepsis, or navel gazing, is a metaphor for contemplating life. Yet, how many of us ever consider the origins of this scar that is universal not only to human beings but to all placental mammals?

The belly button is a remnant of the umbilical cord that served as the baby’s anchor to life during pregnancy by creating a vascular bridge between fetal and maternal circulations. In humans, it is made up of one large vein that carries oxygen-filled blood to the baby, and two arteries that return the oxygen-depleted blood, as well as waste products, to the placenta (Figure 1A). This is the opposite of what happens in the adult body, where arteries carry blood rich in oxygen, and veins return blood depleted of oxygen to the heart, and is due to the dependence of the fetus on the placenta. This reversed scenario is the result of fetal vascular shunts, which provide an alternative path for blood flow at strategic points within the fetus.

**Figure 1.**
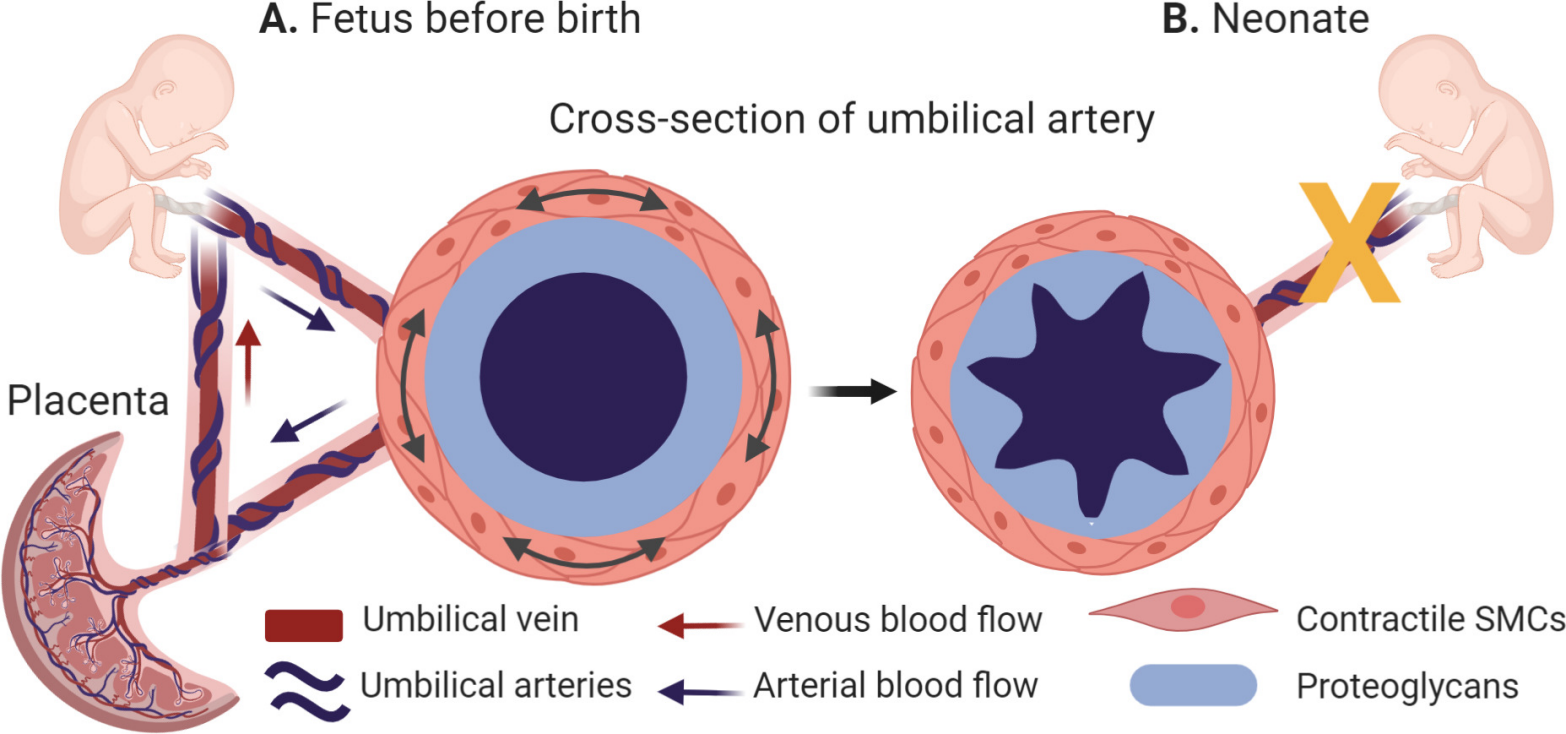
The morphology of the umbilical arteries before and after birth. (**A**) Umbilical arteries (dark blue) transport oxygen and nutrient-depleted blood to the placenta, while the umbilical vein (dark red) carries oxygen and nutrients to the fetus. During pregnancy, the umbilical arteries (cross-section, left side) have a thickened middle layer (the tunica media), whose outer layer is populated with contractile smooth muscle cells (SMCs; orange) and whose inner layer contains a high levels of proteins known as proteoglycans (light blue). (**B**) At birth, the umbilical arteries close rapidly (indicated by the yellow x) to prevent blood loss in the newborn. This is caused by the high local concentrations of proteoglycans, which enable the inner layer of the tunica media to swell and help the smooth muscle cells to contract more strongly (cross section, right side).

​Created with BioRender.com.

Once the baby is born and takes its first breath, a change in oxygen levels in the umbilical arteries prompts the umbilical blood vessels to cease their rhythmic pulsing and the arteries to shut off, a process known as rapid umbilical artery closure (Figure 1B). This is followed by closure of the fetal shunts, thereby establishing the adult circulation. The umbilical vein, on the other hand, remains open for longer. Rapid umbilical artery closure prevents blood loss in the newborn as it detaches from its placenta, and is thought to occur naturally in all placental mammals. However, the mechanism is unclear.

Now, in eLife, Suneel Apte (Cleveland Clinic Lerner Research Institute), Jay Humphrey (Yale University) and colleagues – including Sumeda Nandadasa as first author – report new insights into how the anatomy and physiology of the umbilical vessels facilitate rapid umbilical closure (Nandadasa et al., 2020). The researchers – who are based at institutes in the US, UK and Sweden – used a multi-disciplinary approach to examine the morphological and molecular properties of umbilical blood vessels.

Nandadasa et al. first studied the morphology of the umbilical vessels in humans and mice, and then validated their findings across other placental mammals. In all species, the artery had a thicker middle layer (known as the tunica media) than the vein, and exhibited clear distinctions: the outer layer contained a higher number of smooth muscle cells; and the inner layer contained high levels of two proteins – aggrecan and versican – and low levels of ADAMTS1, an enzyme that cleaves these proteins. Moreover, the lumen of the artery was highly ruffled and partially occluded (Figure 1B). In the vein, the reverse was true: levels of ADAMTS1 were high, and levels of aggrecan and versican were low, and the lumen was much larger.

Aggregan and versican are both proteoglycans located outside of cells that help control the water content in the extracellular environment and provide compressive stiffness to tissues. Aggregan also plays a significant role in the biomechanics of cartilage, but its function in vascular biomechanics has been unclear. However, recent evidence suggests that too much aggregan may contribute to the progression of aneurysms, atherosclerosis and other vascular diseases (Koch et al., 2020). Based on the morphological and molecular properties of the umbilical artery, Nandadasa et al. postulated that prior to rapid umbilical artery closure, high local concentrations of proteoglycans cause the inner layer of the umbilical artery to swell, keeping it open during gestation. Then, at birth, the outer layers of smooth muscle cells contract, causing buckling of the underlying proteoglycan-rich layer and closure of the lumen.

Testing this hypothesis in the mouse model revealed that the smooth muscle cells in the outer wall of the artery were more contractile than those in the vein. Biomechanical tests confirmed that this was caused by the differentiation of the smooth muscle cells (to become more contractile) and by the high levels of proteoglycans, which decreased the distensibility of the arteries. A computational model was used to compare the relative contributions of the morphology of the tunica media, the contractions of smooth muscle cells, and the differential swelling of the umbilical artery, confirming that all three must be present for the artery to close.

Experiments on mice genetically engineered to be deficient in aggrecan and ADAMTS1 revealed that both proteins were needed for the growth of a normal umbilical cord. The results further confirmed that high levels of aggrecan were present when ADAMTS1 was absent, and vice versa. In both cases, the fetuses survived in the womb but died during birth. It could be possible that aggrecan and ADAMTS1 play a role in stillborn births, which are often linked to problems with the placenta and the umbilical cord (Stillbirth Collaborative Research Network Writing Group, 2011).

Mice that lacked ADAMTS1 also suffered from heart problems, furthering the evidence that many cardiovascular defects might be associated with abnormalities of the umbilical vasculature (Kwee et al., 1995; Yang et al., 1995; Oller et al., 2017). Rather than discarding the cord at birth, it may make sense to use it as a sentinel tissue for identifying potential cardiovascular risks later in life.

In summary, the work of Nandadasa et al. highlights the interactions between proteoglycans and smooth muscle cell differentiation, and the resulting biomechanics that facilitate rapid umbilical artery closure. It would be interesting to see if these principles also extend to the fetal shunts (e.g., Hung et al., 2018), or if they could be manipulated to treat diseases affected by blocked arteries. Moreover, they could be used to engineer vessels with a specific composition of proteoglycans to drive the desired differentiation of smooth muscle cells.

## References

[bib1] Hung Y-C, Yeh J-L, Hsu J-H (2018). Molecular mechanisms for regulating postnatal ductus arteriosus closure. International Journal of Molecular Sciences.

[bib2] Koch CD, Lee CM, Apte SS (2020). Aggrecan in cardiovascular development and disease. Journal of Histochemistry & Cytochemistry.

[bib3] Kwee L, Baldwin HS, Shen HM, Stewart CL, Buck C, Buck CA, Labow MA (1995). Defective development of the embryonic and extraembryonic circulatory systems in vascular cell adhesion molecule (VCAM-1) deficient mice. Development.

[bib4] Nandadasa S, Szafron JM, Pathak V, Murtada SI, Kraft CM, O'Donnell A, Norvik C, Hughes C, Caterson B, Domowicz MS, Schwartz NB, Tran-Lundmark K, Veigl M, Sedwick D, Philipson EH, Humphrey JD, Apte SS (2020). Vascular dimorphism ensured by regulated proteoglycan dynamics favors rapid umbilical artery closure at birth. eLife.

[bib5] Oller J, Méndez-Barbero N, Ruiz EJ, Villahoz S, Renard M, Canelas LI, Briones AM, Alberca R, Lozano-Vidal N, Hurlé MA, Milewicz D, Evangelista A, Salaices M, Nistal JF, Jiménez-Borreguero LJ, De Backer J, Campanero MR, Redondo JM (2017). Nitric oxide mediates aortic disease in mice deficient in the metalloprotease Adamts1 and in a mouse model of marfan syndrome. Nature Medicine.

[bib6] Stillbirth Collaborative Research Network Writing Group (2011). Causes of death among stillbirths. Jama.

[bib7] Yang JT, Rayburn H, Hynes RO (1995). Cell adhesion events mediated by alpha 4 integrins are essential in placental and cardiac development. Development.

